# Functional Diversity of Fibroblast Growth Factors in Bone Formation

**DOI:** 10.1155/2015/729352

**Published:** 2015-03-19

**Authors:** Yuichiro Takei, Tomoko Minamizaki, Yuji Yoshiko

**Affiliations:** Department of Calcified Tissue Biology, Hiroshima University Institute of Biomedical & Health Sciences, 1-2-3 Kasumi Minami-ku, Hiroshima 734-8553, Japan

## Abstract

The functional significance of fibroblast growth factor (FGF) signaling in bone formation has been demonstrated through genetic loss-of-function and gain-of-function approaches. FGFs, comprising 22 family members, are classified into three subfamilies: canonical, hormone-like, and intracellular. The former two subfamilies activate their signaling pathways through FGF receptors (FGFRs). Currently, intracellular FGFs appear to be primarily involved in the nervous system. Canonical FGFs such as FGF2 play significant roles in bone formation, and precise spatiotemporal control of FGFs and FGFRs at the transcriptional and posttranscriptional levels may allow for the functional diversity of FGFs during bone formation. Recently, several research groups, including ours, have shown that FGF23, a member of the hormone-like FGF subfamily, is primarily expressed in osteocytes/osteoblasts. This polypeptide decreases serum phosphate levels by inhibiting renal phosphate reabsorption and vitamin D_3_ activation, resulting in mineralization defects in the bone. Thus, FGFs are involved in the positive and negative regulation of bone formation. In this review, we focus on the reciprocal roles of FGFs in bone formation in relation to their local versus systemic effects.

## 1. Introduction

Bone is a connective tissue with a mineralized extracellular matrix that provides support to the body and affects calcium (Ca)/phosphate (inorganic phosphate; Pi) metabolism. Osteoblasts are involved in bone formation via secretion of the organic matrix “osteoid” and the subsequent facilitation of hydroxyapatite crystal formation. Large multinucleated osteoclasts play an active role in bone resorption. Bone formation and resorption, that is, bone metabolism, are regulated by local versus systemic factors. The former includes growth factors and receptor activator of nuclear factor *κ*-*β* ligand (RANKL) and its receptor RANK. Representatives of the latter include parathyroid hormone (PTH), 1*α*,25-dihydroxyvitamin D_3_ (1,25(OH)_2_D_3_), and calcitonin [1]. Growing evidence suggests that additional interactions between bone and extraskeletal organs affect, during development, aging and pathogenesis. For example, undercarboxylated osteocalcin secreted by osteoblasts acts on pancreatic *β*-cells to promote insulin production, which is involved in the regulation of energy metabolism [2]. Osteoblast lineage cells compose hematopoietic [3, 4] and cancer stem cell niches [5], thereby affecting the fates of their stem cells. The adipocyte-derived hormone leptin acts on its specific receptors in the hypothalamus, increases sympathetic activity in bone, and exerts antiosteogenic effects [6]. Serotonin (5-HT) secreted by enterochromaffin cells binds to its receptor 5-HT_2_BR in preosteoblasts and inhibits their proliferation [7]. Further studies in this field are of significance with regard to understanding the precise functions of bone.

Fibroblast growth factors (FGFs) are pleiotropic growth factors that regulate cell proliferation, migration, and differentiation in many organs including bone. Twenty-two family members of FGFs (FGF1–23, wherein FGF15 is the mouse ortholog of human FGF19) have been identified in mammals so far. FGFs can be divided into three subfamilies: canonical, hormone-like, and intracellular [8]. Numerous studies have shown that canonical FGFs, such as FGF2, act in bone. Hormone-like FGF family members are the most recently identified FGFs, and the discovery of these, especially the clinical and experimental studies of FGF23, led us to explore the additional roles of FGFs in bone. Not only FGF23 but also FGF2 is exclusively expressed in osteoblast lineage cells and shares specific receptors (FGF receptors, FGFRs) to transduce intracellular signals, although the effects of these FGFs are variable. The intracellular FGFs, FGF11–14, have been well studied in neurons but not in bone and, therefore, are not discussed here. This review, therefore, provides new insights into the roles of FGFs during bone formation and compares canonical versus hormone-like FGFs.

## 2. The FGF and FGFR Family Members and Their Signaling Pathways

**Figure 1:**
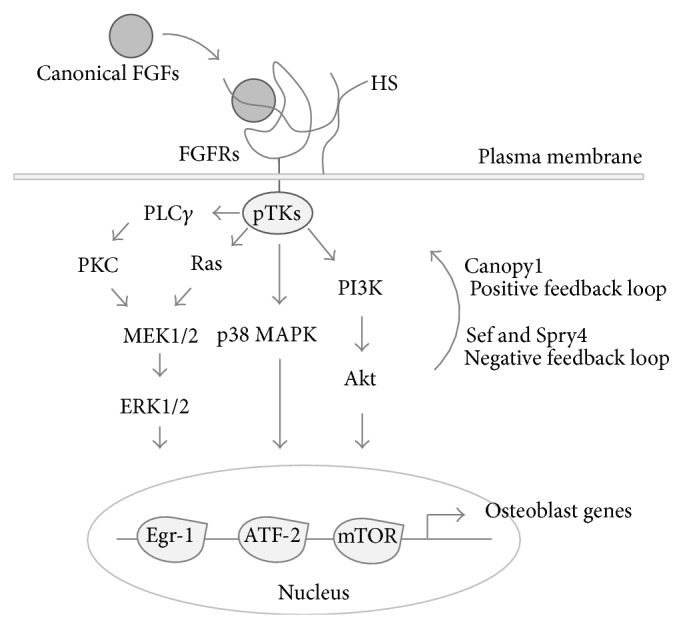
FGF/FGFR signaling and its feedback loops. Ligand-dependent activation of FGFR tyrosine kinases induces ERK1/2, p38 MAPK, and Akt phosphorylation and subsequent upregulation of their downstream transcriptional factors such as early growth response protein-1 (Egr-1), activating transcriptional factor- (ATF-) 2, and mammalian target of rapamycin (mTOR). These transcription factors regulate the expression of genes involved in osteoblastogenesis. Canopy1 acts as positive feedback factor for FGF/FGFR signaling. Sef and Spry4 silence FGF/FGFR signaling. pTKs: phosphorylated tyrosine kinases; PLC*γ*: phospholipase C *γ*; PKC: protein kinase C; MEK: mitogen-activated protein kinase; PI3 K: phosphoinositide 3-kinase.

Canonical FGFs, including FGF2, comprise the most common subfamily that transduces signals through FGFR tyrosine kinases. A heparin-binding domain is conserved among most FGFs, and heparan sulfate (HS) is an integral component for the acquisition of the binding affinity of FGFs to FGFRs. Therefore, these polypeptides can be retained in the extracellular matrix in the vicinity of their secreting cells. Thus, canonical FGFs act as autocrine and/or paracrine factors [10, 11]. The hormone-like subfamily members, FGF15/19, FGF21, and FGF23, contain extra structural features at the C-terminus and require the membrane proteins *α*Klotho/*β*Klotho as cofactors rather than HS to bind to FGFRs [8, 12]. This hallmark difference may pertain to the dynamic properties of the two subfamilies. Both canonical and hormone-like FGFs show their biological activities by activating four distinct FGFRs (also known as the existence of splicing variants “b” and “c” of FGFR1–3) with different binding affinities. For information on the binding affinity of individual FGFs to FGFRs, refer to other reviews and papers (see, e.g., [13]). Many studies have found that tyrosine phosphorylation of the intracellular domain of FGFRs activates the Ras-mitogen-activated protein kinase (MAPK) pathways, including extracellular signal-regulated kinase (ERK)1/2, p38, and c-Jun N-terminal kinase (JNK), the phosphatidylinositol 3-kinase- (PI3 K-) Akt pathway, and the phospholipase C (PLC)*γ*-protein kinase C (PKC) pathway (Figure 1) (see, e.g., [14]). Overall, the spatiotemporal dynamics of FGFs and FGFRs may determine how the FGF family members exert their proper activities in particular cells and tissues.

It is also worth noting that negative and positive modulators expressed in a wide range of cells and tissues play precise roles in FGF signaling, and this may further complicate the functional profiles of FGFs. The sprouty (SPRY) family is a highly conserved group of negative feedback loop modulators of growth factor-mediated MAPK activation that was originally described in* Drosophila* [15]; thereafter, four mammalian orthologs (SPRY1–4) have been identified. Either FGF3 or FGF8 upregulates both mRNA and protein levels of Spry4, while increased Spry4 inhibits both FGF3 and FGF8 signaling by interfering with the downstream activation of FGFR1 in zebrafish blastomeres [16]. Similar expression of* Fgf* genes (*Sef*) encodes a conserved putative transmembrane protein that has sequence similarity with the intracellular domain of the interleukin-17 receptor. This modulator acts as a feedback-induced antagonist of FGF8/Ras/Raf/MAPK signaling in the development of zebrafish embryos [17]. In contrast, Canopy1 (CNPY1) was identified as a positive feedback regulator for FGF-induced signaling [18]. This positive feedback loop between the polypeptide and FGF8/FGFR1 is involved in the cluster formation of dorsal forerunner cells during gastrulation in zebrafish [19]; however, its underlying mechanism in mammals remains to be elucidated.

## 3. Roles of Canonical FGFs on Bone Formation

**Figure 2:**
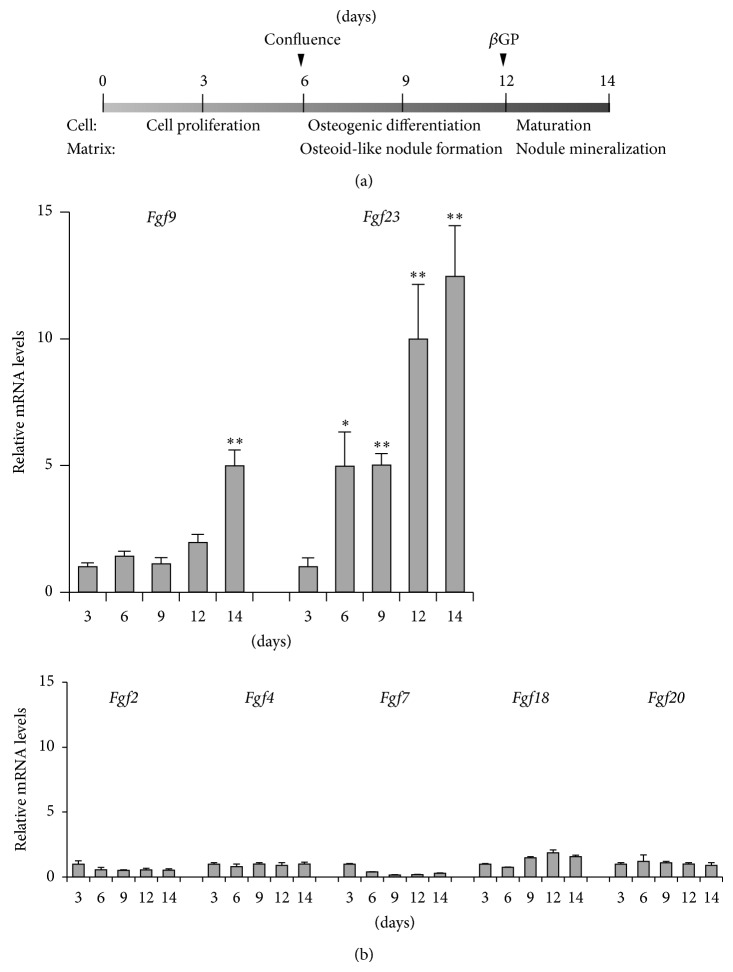
Expression profiling of* Fgf* genes in rat calvaria cell cultures. (a) Outline of osteoblast development. Rat calvaria cells from 21-day-old fetal rats [9] were plated at 3,000 cells per cm^2^ and grown in *α*MEM supplemented with 10% fetal calf serum plus 50 *μ*g/mL ascorbic acid. Cells proliferate, reach confluence at day 6, and subsequently initiate osteoid-like nodule formation. To determine matrix mineralization, 10 mM *β*-glycerophosphate (*β*GP) is added to cultures for 2 days before culture termination. (b) Distinct gene expression patterns of* Fgfs* during osteoblast development. Total RNA was routinely prepared as indicated time points, and cDNA synthesis and quantitative real-time RT PCR (qPCR) were performed using standard protocols. Ribosomal protein L32 was used as internal control. Data represent means ± S.D. *n* = 3. Statistical significance of differences was analyzed with one-way or two-way analysis of variance (ANOVA) with repeated measures, followed by Tukey's multiple comparison test. ^*^ *P* < 0.05 and ^**^ *P* < 0.01 versus day 3.

In addition to our previous data on FGFRs [9], here we show the expression profile of* Fgfs* in a well-established fetal rat calvaria cell model (Figure 2). Among these,* Fgf9* and hormone-like* Fgf23* are abundant and vary in expression levels during osteoblast development.  Table 1 summarizes the primary roles of FGFs in bone formation in multiple models. Human calvaria cell cultures describe, in detail, the roles of FGF2 in osteoblastogenesis [20]. When treated at early developmental stages, FGF2 inhibits alkaline phosphatase (ALP) activity, collagen synthesis, and matrix mineralization and increases cell proliferation; however, when treated at late developmental stages, it has no obvious effects. Because the* in vivo* effects of FGF2 on bone formation are apparent, its potential therapeutic benefit in pediatric surgery and periodontal disease is under consideration [21, 22]. The significant anabolic actions of FGF2 in bone have been widely demonstrated in several animal models; see, for example, growth plate and trabecular bone in growing rats that received daily intravenous injections of FGF2 [23]. Local injections of FGF2 over the calvaria increase new bone formation in mice [24], and those into osteotomized sites of the tibia accelerate surgical fracture repair in rabbits [21]. FGF2 also has an ability to prevent trabecular bone loss in the vertebrae of ovariectomized rats possibly by increasing osteoadipogenic cell proliferation [25].* Fgf2*-null (*Fgf2* ^−/−^ ) mice exhibit a significant decrease of femoral trabecular bone volume and bone formation rate [26]. This can be explained by a downregulation of BMP-2 in* Fgf2* ^−/−^ osteoblasts, resulting in a decrease in ALP activity and nuclear accumulation of the master transcription factor of osteoblastogenesis Runx2 [27]. Furthermore, an inverse correlation between adipogenesis and osteogenesis is observed in* Fgf* ^**−/−^ mice, and FGF2 blocks adipocyte formation and increases ALP-positive colony formation in bone marrow cell cultures independent of FGF2 [28]. In FGF2, most attention has been dedicated to the smallest 18-kDa variant (LMW). In addition, genetic manipulation of LMW FGF2 in skeletal tissues contributes to bone phenotypes* in vivo* [29]. However, there are several higher molecular weight (HMW) variants of the polypeptide. Additional information on the representative roles of the HMW variants in bone is shown below.

**Figure 3:**
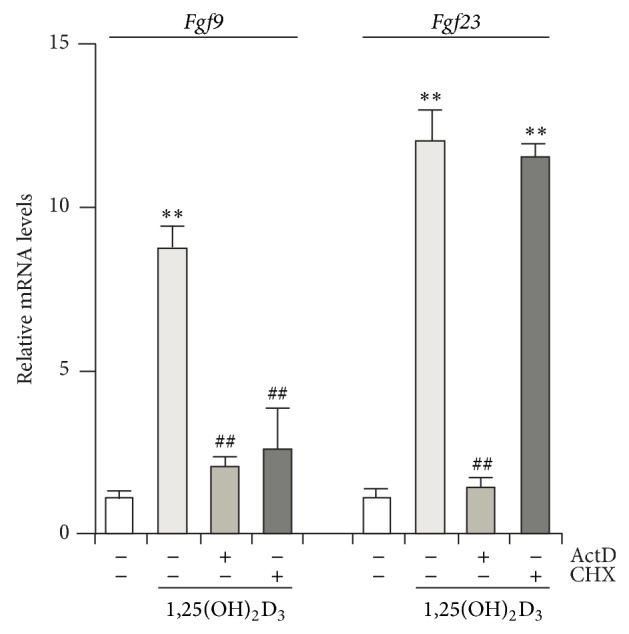
1,25(OH)_2_D_3_ increases* Fgf9* and* Fgf23* gene expression at late development stages in rat calvaria cell cultures. Rat calvaria cells were obtained as shown in Figure 2. At day 11, nodule-forming cells were stripped by collagenase and replated (subcultures). Four days later, osteoblast subcultures were pretreated with or without actinomycin D (ActD) or cycloheximide (CHX), followed by incubation with 1 nM 1,25(OH)_2_D_3_ for 6 h. See the above mentioned for qPCR. Data represent means ± S.D. *n* = 3. Statistical significance of differences was analyzed with one-way or two-way analysis of variance (ANOVA) with repeated measures, followed by Tukey's multiple comparison test. ^**^ *P* < 0.01 versus vehicle alone; ^##^ *P* < 0.01 versus 1,25(OH)_2_D_3_ alone.

Compared with FGF2, other canonical FGFs have not been studied in detail (Table 1). Although* Fgf1* expression was not obvious in our model, its transcript appears to act in the same manner as FGF2 [30]. Intravenous administration of FGF1 increases bone formation of femoral diaphysis in normal rats [30] and tibial metaphysis in ovariectomized rats [24]. However,* Fgf1* ^−/−^ mice do not display any gross phenotypic defects [31]. Because deficiency of FGF1 in mice exacerbated high-fat diet-induced diabetic phenotypes, such as insulin resistance and defects in adipose remodeling in gonadal white adipose tissue, FGF1, may directly and/or indirectly act on bone. FGF4 is more specific to mesenchymal cells, but its subcutaneous injections increase trabecular bone mineral density in the mouse femur [32]. Much less is known about the roles of FGF6 [33], FGF7 [34], and FGF8 [35] in bone; the expression of* Fgf7* but not of* Fgf6* and* Fgf8* is detected in our calvaria cell model, and FGF6 shows catabolic effects on osteoblastic cells, but others have anabolic function* in vitro*. Histological evidence for chondrogenesis with the upregulation of the* Sox9* and* Col2a1* genes is seen in cranial mesenchymal cells of transgenic mice overexpressing FGF9, suggesting that FGF9 converts intramembranous ossification to endochondral ossification [36]. FGF9 also shows supportive effects on FGF2-dependent trabecular bone formation [37]. Among* Fgfs* expressed in our model,* Fgf9* is abundant during the late developmental stages, along with* Fgf23* levels (Figure 2). Notably, both mRNA levels are upregulated by 1,25(OH)_2_D_3_, while only* Fgf9 *levels are suppressed by pretreatment of cycloheximide, a protein synthesis inhibitor, as well as the transcriptional inhibitor actinomycin D (Figure 3). Thus, 1,25(OH)_2_D_3_-dependent expression of* Fgf9* but not* Fgf23* may result from* de novo* protein synthesis. Additional role(s) and the precise regulatory mechanism of FGF9 in osteoblast functions remain to be elucidated. Functional anomalies in FGF10 signals may be involved in craniosynostosis [38], but there are no obvious effects of FGF10 in our rat (unpublished data) and mouse calvaria cells [39]. Treatment of mouse calvaria cells with FGF18 promotes proliferation and suppresses differentiation and matrix mineralization [39]. In* Fgf18* ^−/−^ mouse embryos, calvaria cell proliferation and bone mineralization and kyphosis are observed in the cervical and upper thoracic spine [40]. Together with the observation that treatment of mouse calvaria cells with FGF18 increases proliferation and decreases matrix mineralization [39], the effects of this polypeptide on bone formation appear to be similar to those of FGF2.

## 4. Physiological and Pathological Importance of FGFRs in Bone 

The dynamics of FGFRs are also an important determinant of FGF-mediated bone formation. Indeed, mutations in FGFR1 and FGFR2 account for the craniosynostosis and chondrodysplasia syndromes in humans [41–44], suggesting that both FGFRs are important for endochondral and intramembranous bone formation. Because* Fgfr1* ^−/−^ mice are embryonic lethal shortly after gastrulation [45], osteochondrocyte lineage- and osteoblast-specific FGFR1 knockout mice were generated under the control of the* pro*α*1(II) collagen (Col2)* and* pro*α*1(I) collagen (Col1)* promoters, respectively.* Col2*-mediated FGFR1 inactivation delays chondrocyte and osteoblast maturation, while* Col1*-dependent FGFR1 deficiency accelerates osteoblast differentiation with stimulated mineral deposition and reduces osteoclast activity [46]. Gain-of-function missense mutations in* Fgfr2* (S252W and P253R) cause craniosynostosis syndromes, including Crouzon and Apert syndromes [47, 48]. Indeed, heterozygous* Fgfr2* (S252W) mutant mice show midline sutural bone defects and craniosynostosis with abnormal osteoblastic proliferation and differentiation [49]. An* in vitro* study shows that constitutively active FGFR2 (S252W) induces the ERK1/2 and PKC pathways causing osteoblastic differentiation in the murine mesenchymal cell line C3H10T1/2 [50]. Three of the* Fgfr3* gain-of-function mutations have been reported to cause chondrodysplasia and craniosynostosis. Achondroplasia, the most common form of human dwarfism, is associated with the G380R mutation [51]. The P250R mutation causes Muenke syndrome, a common syndrome of craniosynostosis [52]. Crouzon syndrome and acanthosis nigricans, a skin pigmentation disorder, result from the A391E mutation [53]. Unlike FGFR1 and FGFR2 deficient mice, systemic* Fgfr3* null mice are viable and show progressive osteodysplasia with expanded growth plate cartilage [54]. Taken together, because FGF9, a preferred ligand for FGFR3, upregulates* osteopontin* (*Opn*) in chicken chondrocytes [55], FGFR3 signaling may affect chondrocytes rather than osteoblasts [54]. In contrast to these three FGFRs, there are quite a few reports about the relationship between FGFR4 and bone formation. Cool et al. indicated that FGFR4 is expressed in preosteoblasts and osteoblasts in neonatal mouse calvaria, suggesting that FGFR4 is involved in osteogenesis [56], but its role in bone remains unclear.

## 5. FGF23 and FGF19 Subfamily Members as Hormone-Like Factors

FGF23 is the last member of the FGF family, and its significant roles in Pi and vitamin D metabolism are obvious in genetically engineered mice [57–59] (also see review [60]). FGF23 was originally discovered as the gene responsible for autosomal dominant hypophosphatemic rickets [61] and thereafter as a phosphaturic factor produced by mesenchymal tumors in tumor-induced osteomalacia [62]. FGF23 is predominately expressed in osteoblasts/osteocytes [63–66]. Type I transmembrane protein *α*Klotho acts as a coreceptor for FGF23 to convert canonical FGFRs (FGFR1c, FGFR3c, and FGFR4) into a specific receptor for FGF23 [67, 68]. Therefore, organs expressing *α*Klotho, such as the kidney, parathyroid glands, and choroid plexus, appear to be targets of FGF23 [69]. FGF23 decreases the expression of renal type II sodium-phosphate cotransporters (*Slc34a1* and* Slc34a3*) and 25-hydroxyvitamin D_3_ (25(OH)D_3_) 1*α*-hydroxylase, resulting in a decrease in serum Pi and 1,25(OH)_2_D_3_ levels, respectively, in mice and rats [70, 71]. Meanwhile, 1,25(OH)_2_D_3_ induces* Fgf23* expression in rat osteosarcoma ROS17/2.8 cells [72] as well as our rat calvaria cells [73]. Together with the result that intraperitoneal injections of 1,25(OH)_2_D_3_ into mice increase serum FGF23 levels, there seems to be a feedback loop between FGF23 and 1,25(OH)_2_D_3_ [72]. FGF23 also decreases the expression of PTH [74], although this is not simply regulated by the FGF23-*α*Klotho axis [75]. Transgenic mice expressing constitutively active PTHR1 in osteocytes exhibit increased serum FGF23 levels independently of serum Ca and Pi levels and* Fgf23* expression in osteoblasts and osteocytes [76]. Comparison of* Fgfr1/3/4* single and double knockout mice indicates that FGFR1 and FGFR3/4 may be involved in renal Pi reabsorption [70] and vitamin D metabolism [77], respectively. Additional factors, for example, Pi [78], sympathetic activation [79], and circulating *α*Klotho [80], may be involved in FGF23 expression/production; however, the regulation of FGF23 expression is still under investigation.

**Figure 4:**
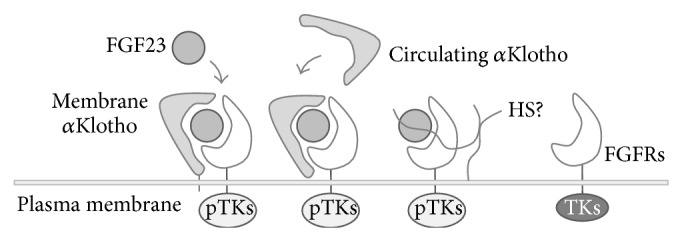
Possible klotho-dependent and klotho-independent mechanisms of FGF23 actions. FGF23 may activate FGFR tyrosine kinases with or without membrane and circulating *α*Klotho. TKs: nonphosphorylated tyrosine kinases.

Both of ectopic (hepatic) overexpression and osteoblast/osteocyte-specific overexpression of the* Fgf23* transgene result in lower bone mineral density of the femur with hypophosphatemia and high serum levels of PTH [57, 58]. The lack of either FGF23 or *α*Klotho causes aberrant Ca/Pi and vitamin D metabolism, thus ensuring skeletal anomalies and ectopic calcification [59, 81, 82].* Fgf23* ^−/−^/*Opn* ^−/−^ double-knockout (DKO) mice mimic hyperphosphatemia in* Fgf23* ^−/−^ mice, but the severe osteoidosis in* Fgf23* ^−/−^ is markedly reduced [83].* Fgf23* ^−/−^/*Slc34a1* ^−/−^ DKO mice reverse hyper- to hypophosphatemia in keeping with hypomineralization in bone [84]. These observations suggest that skeletal anomalies that involve FGF23 may result not only from serum Pi levels but also from intrinsic anomalies in bone. FGF23 may act independently of the membrane protein *α*Klotho (Figure 4). For example, overexpression of FGF23 in cultured rat calvaria cells impairs osteoblast differentiation and mineralized matrix formation but not mineralization, via activation of FGFR1 [9]. One plausible explanation is that the existence of the soluble form (circulating *α*Klotho) shedding from the extracellular domain of *α*Klotho [85, 86] may act as a cofactor for FGF23. In fact, effects of FGF23 in MC3T3-E1 cells (a mouse osteoblastic cell line) cultured with circulating *α*Klotho [87] mimic the results observed in rat calvaria cells [9]. In mouse chondrocytes, FGF23 activates FRS2*α*, FGFR substrate 2*α*, and ERK1/2, resulting in a decrease in chondrocyte proliferation in the presence of circulating *α*Klotho [88]. In contrast, *α*Klotho is not required for FGF23 action in some cells. For instance, FGF23 can induce the hypertrophy of neonatal rat ventricular cardiomyocytes, in which *α*Klotho is not detected [89]. In addition, FGF23 decreases PTH secretion in thyroparathyroid organ cultures from parathyroid-specific *α*Klotho-deficient mice [75]. It is still unknown why FGF23 targets the kidney and parathyroid glands, even in the presence of circulating *α*Klotho and/or the ubiquitous expression of FGFRs.

The roles of two other members of the hormone-like FGF19 subfamily, FGF19 and FGF21, in bone formation remain to be elucidated.* Fgf19* transcripts are predominantly expressed in the ileum, while* Fgf21* mRNA is expressed in the liver, pancreas, and white adipose tissue [90]. In skeletal tissue under normal conditions, FGF19, but not FGF21, is also detectable at the protein level in human fetal growth plate cartilage [91]. Interestingly, the treatment of mouse bone marrow cells with FGF21 increases *βKlotho* and* Fgf21* mRNA expression, especially in the presence of rosiglitazone [92], an agonist of the master regulator for adipogenesis, PPAR*γ*, possibly affecting bone formation. Thus, genetic FGF21 loss and gain of function in mice increase and decrease bone mass [92], respectively, suggesting that FGF21/*β*Klotho may act as an inhibitor of bone formation.

## 6. Local and Systemic Effects of FGFs during Bone Formation, Focusing on FGF2, FGF21, and FGF23

As above, FGF2 and FGF23 may exhibit distinct activities during different stages of osteoblast differentiation, such as cell proliferation versus matrix (osteoid) mineralization. In contrast to osteogenic cell proliferation, differentiation, and associated matrix formation, the molecular mechanism(s) underlying matrix mineralization remains to be fully elucidated. Human FGF2 has multiple isoforms via an alternative initiation of translation at CUG codons from a single* FGF2* gene: LMW and high (HMW FGF2, 22-kDa, 22.5-kDa, 24-kDa, and 34-kDa) molecular forms [93]. LMW FGF2—exactly the same FGF2 as described above—is predominantly expressed in osteoblast precursors and activates intracellular signaling via FGFR in an autocrine/paracrine manner. While recent evidence indicates that extracellular LMW FGF2 can translocate to the nucleus after internalization [94], there is little evidence for this process in bone to date. The HMW FGF2 isoforms are not released from the cells and localized to the nucleus and regulate gene expression to exert specific effects. Transgenic mice overexpressing human HMW FGF2 (22-kDa, 23-kDa, and 24-kDa) under the* Col1* promoter (*Col3.6*) exhibit lower bone mineral density with decreased bone formation and increased bone resorption [95]. Interestingly, upregulation of* Fgf23* expression and hypophosphatemia are observed in these mice [95]. These observations may lead to the development of an additional framework for understanding the effects of the HMW FGF2 and FGF23 on bone mineralization.

It is well known that elevated serum FGF23 levels are the most common predictor in patients with chronic kidney disease [96]. Serum FGF23 levels are positively correlated to aortic arterial calcification in hemodialysis patients [97]. Recent studies demonstrate that FGF23 exacerbates left ventricle hypertrophy where *α*Klotho might not be expressed [89] and elevated plasma FGF23 levels are associated with low body mass index and dyslipidemia in dialysis patients [98]. Thus, systemic actions of FGF23 may reach organs dependently and independently of *α*Klotho. Although skeletal tissues do not express* Fgf21* under normal conditions, circulating FGF21 seems to suppress osteoblastogenesis and induce adipogenesis [92]. Also, FGF21 itself enhances* Fgf21* and *βKlotho* expression in bone marrow-derived adipocytes, and increases in FGF21 and *β*Klotho have a synergetic effect on its signaling in local area [92]. Comprehensive analyses are needed to determine the local versus systemic effects of FGF21 on bone. Taken all together, FGFs expressed in bone are involved in bone formation directly and indirectly, which indicates that FGFs mediate the interrelationships between bone and other organs under normal and/or clinical situations. The clinical importance of FGF23/21 is now becoming clearer owing to the recent findings in FGF research. However, precise elucidation of FGF mechanisms is still required.

## 7. Conclusion

The skeleton is a multipotent organ that is fundamental for the survival of vertebrates. Bone and mineral homeostasis are strictly controlled by multiple mechanisms including FGF/FGFR signaling. Canonical and hormone-like FGFs regulate bone formation at different developmental stages in different ways, and these members may compensate for one another in bone and/or extraskeletal tissues. In order to understand these mechanisms, the balance between local and systemic regulation needs to be considered.

## Figures and Tables

**Table 1:** Roles of FGFs in bone.

FGFs		Models				Outcomes	Animals	Ref.
		*In vivo *		*Ex vivo *	*In vitro *			
Types	Members	Genetic manipulations	Recombinant proteins		Recombinant proteins and so forth			
Canonical	FGF1	Systemic deletion				No obvious effects	Mice	[31]
			Injections over the calvaria			Osteoblastic cell proliferation and new bone formation ↑	Mice	[24]
			Intravenous injections			Prevention of the ovariectomized (OVX)-related bone loss	OVX Rats	
					Osteoblasts	Cell proliferation ↑, but nodule formation and mineralization ↓	Rats	[30]
	FGF2	Systemic deletion		Calvaria cells		Cell proliferation ↓	Mice	[26]
				Bone marrow cells		ALP-positive colonies and mineralized nodules ↓	Mice	[26, 28]
						Trabecular bone ↓		
		Osteoblast-specific expression of human HMW FGF2				Dwarfism, osteomalacia, none mineral density ↓, serum phosphate levels, and FGF23 expression ↑	Mice	[95]
		
		Osteoblast-specific expression of human LMW FGF2		Bone marrow cells		ALP-positive colonies and mineralized nodules ↑	Mice	[29]
						Increased bone formation ↑, sFRP-1 expression ↓		
		Systemic deletion of human LMW FGF2		Bone marrow cells		Reverse effects as above		
		
			Intravenous injections			Growth plate width and trabecular bone ↑ and periosteal bone ↓	Rats	[23]
			Subcutaneously injections			Osteoid volume in lumbar vertebra ↑	OVX Rats	[25]
			Injections over the calvaria			Osteoblastic cell proliferation and new bone formation ↑	Mice	[24]
			Single local injection into the distracted callus			Bone formation in the callotasis model ↑	Rabbits	[21]
			Subcutaneous transplantations of human bone marrow cells treated with FGF2			New bone formation in trabecular bone ↑	Nude mice	[37]
					Bone marrow cells	Cell proliferation and matrix mineralization ↑	Humans	
					Calvaria cells	Cell proliferation ↑, matrix mineralization ↓	Mice	[39]
						Differentiation stage-specific effects; cell proliferation ↑,	Humans	[20]
						Osteogenic differentiation ↓ in less mature cells		
						Matrix mineralization ↑ in more mature cells		
					Osteoblasts from trabecular bone	Cell proliferation ↑, ALP activity, and matrix mineralization ↓	Humans	[33]
	FGF4		Subcutaneous injections			Bone formation ↑	Mice	[32]
	FGF6				Osteoblasts from trabecular bone	Cell proliferation ↑, ALP activity, and matrix mineralization ↓	Humans	[33]
	FGF7				Embryonic stem cells	Mineralized nodules and osteoblast marker gene expression ↑	Mice	[34]
	FGF8				Osteogenic ROB-26 cells	ALP activity and *Runx2 *expression ↑	Rats	[35]
	FGF9		Subcutaneous transplantations of human bone marrow cells treated with FGF2 plus FGF9			Effect of FGF2 on new bone formation in trabecular bone ↑	Nude mice	[37]
					Bone marrow cells	Effect of FGF2 on cell proliferation and mineralization ↑	Humans	
	FGF10	Systemic deletion in *Fgfr2 *mutant mice				Rescue of craniosynostosis and skeletal defects	Mice	[38]
					Calvaria cells	No obvious effects	Mice	[39]
	FGF18	Systemic deletion				Skeletal defects, proliferation of osteogenic cells, and maturation of osteoblasts ↓	Mice	[40]
					Calvaria cells	Cell proliferation ↑, matrix mineralization ↓	Mice	[39]

Hormone-like	FGF21	Overexpression				Trabecular bone ↓	Mice	[92]
		Systemic deletion				Reverse effects as above		
	FGF23	Systemic deletion				Bone mineralization ↓ with hyperphosphatemia	Mice	[81]
		Overexpression				Bone abnormality with hypophosphatemia and serum PTH levels ↑	Mice	[57]
		Osteoblast-specific overexpression of FGF23					Mice	[58]
					Calvaria cells with adenoviral FGF23 overexpression	Osteogenic differentiation and matrix mineralization ↓	Rats	[9]
					Osteoblastic MC3T-E1 cells	Cell proliferation ↑, matrix mineralization ↓	Mice	[87]

↑: increase; ↓: decrease. Ref.: References.

## References

[1] Feng X., McDonald J. M. (2011). Disorders of bone remodeling. *Annual Review of Pathology*.

[2] Ferron M., McKee M. D., Levine R. L., Ducy P., Karsenty G. (2012). Intermittent injections of osteocalcin improve glucose metabolism and prevent type 2 diabetes in mice. *Bone*.

[3] Calvi L. M., Adams G. B., Weibrecht K. W. (2003). Osteoblastic cells regulate the haematopoietic stem cell niche. *Nature*.

[4] Zhang J., Niu C., Ye L. (2003). Identification of the haematopoietic stem cell niche and control of the niche size. *Nature*.

[5] Iwasaki H., Suda T. (2009). Cancer stem cells and their niche. *Cancer Science*.

[6] Takeda S., Elefteriou F., Levasseur R. (2002). Leptin regulates bone formation via the sympathetic nervous system. *Cell*.

[7] Collet C., Schiltz C., Geoffroy V., Maroteaux L., Launay J.-M., de Vernejoul M.-C. (2008). The serotonin 5-HT_2B_ receptor controls bone mass via osteoblast recruitment and proliferation. *The FASEB Journal*.

[8] Kuro-o M. (2008). Endocrine FGFs and Klothos: emerging concepts. *Trends in Endocrinology & Metabolism*.

[9] Wang H., Yoshiko Y., Yamamoto R. (2008). Overexpression of fibroblast growth factor 23 suppresses osteoblast differentiation and matrix mineralization in vitro. *Journal of Bone and Mineral Research*.

[10] Böttcher R. T., Niehrs C. (2005). Fibroblast growth factor signaling during early vertebrate development. *Endocrine Reviews*.

[11] Thisse B., Thisse C. (2005). Functions and regulations of fibroblast growth factor signaling during embryonic development. *Developmental Biology*.

[12] Kurosu H., Kuro-O M. (2009). Endocrine fibroblast growth factors as regulators of metabolic homeostasis. *BioFactors*.

[13] Mohammadi M., Olsen S. K., Ibrahimi O. A. (2005). Structural basis for fibroblast growth factor receptor activation. *Cytokine and Growth Factor Reviews*.

[14] Dailey L., Ambrosetti D., Mansukhani A., Basilico C. (2005). Mechanisms underlying differential responses to FGF signaling. *Cytokine and Growth Factor Reviews*.

[15] Hacohen N., Kramer S., Sutherland D., Hiromi Y., Krasnow M. A. (1998). *Sprouty* encodes a novel antagonist of FGF signaling that patterns apical branching of the *Drosophila* airways. *Cell*.

[16] Fürthauer M., Reifers F., Brand M., Thisse B., Thisse C. (2001). *Sprouty4* acts in vivo as a feedback-induced antagonist of FGF signaling in zebrafish. *Development*.

[17] Fürthauer M., Lin W., Ang S.-L., Thisse B., Thisse C. (2002). Sef is a feedback-induced antagonist of RAs/MAPK-mediated FGF signalling. *Nature Cell Biology*.

[18] Hirate Y., Okamoto H. (2006). Canopy1, a novel regulator of FGF signaling around the midbrain-hindbrain boundary in zebrafish. *Current Biology*.

[19] Matsui T., Thitamadee S., Murata T. (2011). Canopy1, a positive feedback regulator of FGF signaling, controls progenitor cell clustering during Kupffer's vesicle organogenesis. *Proceedings of the National Academy of Sciences of the United States of America*.

[20] Debiais F., Hott M., Graulet A. M., Marie P. J. (1998). The effects of fibroblast growth factor-2 on human neonatal calvaria osteoblastic cells are differentiation stage specific. *Journal of Bone and Mineral Research*.

[21] Okazaki H., Kurokawa T., Nakamura K., Matsushita T., Mamada K., Kawaguchi H. (1999). Stimulation of bone formation by recombinant fibroblast growth factor-2 in callotasis bone lengthening of rabbits. *Calcified Tissue International*.

[22] Kitamura M., Akamatsu M., MacHigashira M. (2011). FGF-2 stimulates periodontal regeneration: results of a multi-center randomized clinical trial. *Journal of Dental Research*.

[23] Nagai H., Tsukuda R., Mayahara H. (1995). Effects of basic fibroblast growth factor (bFGF) on bone formation in growing rats. *Bone*.

[24] Dunstan C. R., Boyce R., Boyce B. F. (1999). Systemic administration of acidic fibroblast growth factor (FGF-1) prevents bone loss and increases new bone formation in ovariectomized rats. *Journal of Bone and Mineral Research*.

[25] Aguirre J. I., Leal M. E., Rivera M. F., Vanegas S. M., Jorgensen M., Wronski T. J. (2007). Effects of basic fibroblast growth factor and a prostaglandin E2 receptor subtype 4 agonist on osteoblastogenesis and adipogenesis in aged ovariectomized rats. *Journal of Bone and Mineral Research*.

[26] Montero A., Okada Y., Tomita M. (2000). Disruption of the fibroblast growth factor-2 gene results in decreased bone mass and bone formation. *Journal of Clinical Investigation*.

[27] Naganawa T., Xiao L., Coffin J. D. (2008). Reduced expression and function of bone morphogenetic protein-2 in bones of *Fgf2* null mice. *Journal of Cellular Biochemistry*.

[28] Xiao L., Sobue T., Esliger A. (2010). Disruption of the *Fgf2* gene activates the adipogenic and suppresses the osteogenic program in mesenchymal marrow stromal stem cells. *Bone*.

[29] Xiao L., Liu P., Li X. (2009). Exported 18-kDa isoform of fibroblast growth factor-2 is a critical determinant of bone mass in mice. *Journal of Biological Chemistry*.

[30] Tang K. T., Capparelli C., Stein J. L. (1996). Acidic fibroblast growth factor inhibits osteoblast differentiation in vitro: altered expression of collagenase, cell growth-related, and mineralization-associated genes. *Journal of Cellular Biochemistry*.

[31] Miller D. L., Ortega S., Bashayan O., Basch R., Basilico C. (2000). Compensation by fibroblast growth factor 1 (FGF1) does not account for the mild phenotypic defects observed in FGF2 null mice. *Molecular and Cellular Biology*.

[32] Kuroda S., Kasugai S., Oida S., Iimura T., Ohya K., Ohyama T. (1999). Anabolic effect of aminoterminally truncated fibroblast growth factor 4 (FGF4) on bone. *Bone*.

[33] Bosetti M., Leigheb M., Brooks R. A., Boccafoschi F., Cannas M. F. (2010). Regulation of osteoblast and osteoclast functions by FGF-6. *Journal of Cellular Physiology*.

[34] Jeon Y.-M., Kook S.-H., Rho S.-J. (2013). Fibroblast growth factor-7 facilitates osteogenic differentiation of embryonic stem cells through the activation of ERK/Runx2 signaling. *Molecular and Cellular Biochemistry*.

[35] Omoteyama K., Takagi M. (2009). FGF8 regulates myogenesis and induces Runx2 expression and osteoblast differentiation in cultured cells. *Journal of Cellular Biochemistry*.

[36] Govindarajan V., Overbeek P. A. (2006). FGF9 can induce endochondral ossification in cranial mesenchyme. *BMC Developmental Biology*.

[37] Kizhner T., Ben-David D., Rom E., Yayon A., Livne E. (2011). Effects of FGF2 and FGF9 on osteogenic differentiation of bone marrow-derived progenitors. *In Vitro Cellular and Developmental Biology—Animal*.

[38] Hajihosseini M. K., Duarte R., Pegrum J. (2009). Evidence that Fgf10 contributes to the skeletal and visceral defects of an apert syndrome mouse model. *Developmental Dynamics*.

[39] Shimoaka T., Ogasawara T., Yonamine A. (2002). Regulation of osteoblast, chondrocyte, and osteoclast functions by fibroblast growth factor (FGF)-18 in comparison with FGF-2 and FGF-10. *The Journal of Biological Chemistry*.

[40] Ohbayashi N., Shibayama M., Kurotaki Y. (2002). FGF18 is required for normal cell proliferation and differentiation during osteogenesis and chondrogenesis. *Genes and Development*.

[41] Chen L., Deng C.-X. (2005). Roles of FGF signaling in skeletal development and human genetic diseases. *Frontiers in Bioscience*.

[42] Morriss-Kay G. M., Wilkie A. O. M. (2005). Growth of the normal skull vault and its alteration in craniosynostosis: insights from human genetics and experimental studies. *Journal of Anatomy*.

[43] Ornitz D. M., Marie P. J. (2002). FGF signaling pathways in endochondral and intramembranous bone development and human genetic disease. *Genes & Development*.

[44] Ornitz D. M. (2005). FGF signaling in the developing endochondral skeleton. *Cytokine and Growth Factor Reviews*.

[45] Yamaguchi T. P., Harpal K., Henkemeyer M., Rossant J. (1994). *fgfr*-1 is required for embryonic growth and mesodermal patterning during mouse gastrulation. *Genes and Development*.

[46] Jacob A. L., Smith C., Partanen J., Ornitz D. M. (2006). Fibroblast growth factor receptor 1 signaling in the osteo-chondrogenic cell lineage regulates sequential steps of osteoblast maturation. *Developmental Biology*.

[47] Jabs E. W., Li X., Scott A. F. (1994). Jackson-Weiss and Crouzon syndromes are allelic with mutations in fibroblast growth factor receptor 2. *Nature Genetics*.

[48] Wilkie A. O. M., Slaney S. F., Oldridge M. (1995). Apert syndrome results from localized mutations of FGFR2 and is allelic with Crouzon syndrome. *Nature Genetics*.

[49] Wang Y., Xiao R., Yang F. (2005). Abnormalities in cartilage and bone development in the Apert syndrome FGFR2^+/S252W^ mouse. *Development*.

[50] Miraoui H., Oudina K., Petite H., Tanimoto Y., Moriyama K., Marie P. J. (2009). Fibroblast growth factor receptor 2 promotes osteogenic differentiation in mesenchymal cells via ERK1/2 and protein kinase C signaling. *The Journal of Biological Chemistry*.

[51] Rousseau F., Bonaventure J., Legeai-Mallet L. (1994). Mutations in the gene encoding fibroblast growth factor receptor-3 in achondroplasia. *Nature*.

[52] Muenke M., Gripp K. W., McDonald-McGinn D. M. (1997). A unique point mutation in the fibroblast growth factor receptor 3 gene (FGFR3) defines a new craniosynostosis syndrome. *The American Journal of Human Genetics*.

[53] Meyers G. A., Orlow S. J., Munro I. R., Przylepa K. A., Jabs E. W. (1995). Fibroblast growth factor receptor 3 (FGFR3) transmembrane mutation in Crouzon syndrome with acanthosis nigricans. *Nature Genetics*.

[54] Deng C., Wynshaw-Boris A., Zhou F., Kuo A., Leder P. (1996). Fibroblast growth factor receptor 3 is a negative regulator of bone growth. *Cell*.

[55] Weizmann S., Tong A., Reich A., Genina O., Yayon A., Monsonego-Ornan E. (2005). FGF upregulates osteopontin in epiphyseal growth plate chondrocytes: implications for endochondral ossification. *Matrix Biology*.

[56] Cool S., Jackson R., Pincus P., Dickinson I., Nurcombe V. (2002). Fibroblast growth factor receptor 4 (FGFR4) expression in newborn murine calvaria and primary osteoblast cultures. *International Journal of Developmental Biology*.

[57] Bai X., Miao D., Li J., Goltzman D., Karaplis A. C. (2004). Transgenic mice overexpressing human fibroblast growth factor 23 (R176Q) delineate a putative role for parathyroid hormone in renal phosphate wasting disorders. *Endocrinology*.

[58] Larsson T., Marsell R., Schipani E. (2004). Transgenic mice expressing fibroblast growth factor 23 under the control of the *α*1(I) collagen promoter exhibit growth retardation, osteomalacia, and disturbed phosphate homeostasis. *Endocrinology*.

[59] Shimada T., Kakitani M., Yamazaki Y. (2004). Targeted ablation of *Fgf23* demonstrates an essential physiological role of FGF23 in phosphate and vitamin D metabolism. *The Journal of Clinical Investigation*.

[60] Quarles L. D. (2012). Skeletal secretion of FGF-23 regulates phosphate and vitamin D metabolism. *Nature Reviews Endocrinology*.

[61] White K. E., Evans W. E., O'Riordan J. L. H. (2000). Autosomal dominant hypophosphataemic rickets is associated with mutations in FGF23. *Nature Genetics*.

[62] Shimada T., Mizutani S., Muto T. (2001). Cloning and characterization of FGF23 as a causative factor of tumor-induced osteomalacia. *Proceedings of the National Academy of Sciences of the United States of America*.

[63] Yoshiko Y., Wang H., Minamizaki T. (2007). Mineralized tissue cells are a principal source of FGF23. *Bone*.

[64] Mirams M., Robinson B. G., Mason R. S., Nelson A. E. (2004). Bone as a source of FGF23: regulation by phosphate?. *Bone*.

[65] Masuyama R., Stockmans I., Torrekens S. (2006). Vitamin D receptor in chondrocytes promotes osteoclastogenesis and regulates FGF23 production in osteoblasts. *The Journal of Clinical Investigation*.

[66] Liu S., Tang W., Zhou J., Vierthaler L., Quarles L. D. (2007). Distinct roles for intrinsic osteocyte abnormalities and systemic factors in regulation of FGF23 and bone mineralization in Hyp mice. *The American Journal of Physiology—Endocrinology and Metabolism*.

[67] Urakawa I., Yamazaki Y., Shimada T. (2006). Klotho converts canonical FGF receptor into a specific receptor for FGF23. *Nature*.

[68] Kurosu H., Ogawa Y., Miyoshi M. (2006). Regulation of fibroblast growth factor-23 signaling by Klotho. *Journal of Biological Chemistry*.

[69] Imura A., Tsuji Y., Murata M. (2007). *α*-klotho as a regulator of calcium homeostasis. *Science*.

[70] Gattineni J., Bates C., Twombley K. (2009). FGF23 decreases renal NaPi-2a and NaPi-2c expression and induces hypophosphatemia in vivo predominantly via FGF receptor 1. *The American Journal of Physiology—Renal Physiology*.

[71] Saito H., Kusano K., Kinosaki M. (2003). Human fibroblast growth factor-23 mutants suppress Na^+^-dependent phosphate co-transport activity and 1*α*,25-dihydroxyvitamin D_3_ production. *The Journal of Biological Chemistry*.

[72] Liu S., Tang W., Zhou J. (2006). Fibroblast growth factor 23 is a counter-regulatory phosphaturic hormone for vitamin D. *Journal of the American Society of Nephrology*.

[73] Yamamoto R., Minamizaki T., Yoshiko Y. (2010). 1*α*,25-dihydroxyvitamin D_3_ acts predominately in mature osteoblasts under conditions of high extracellular phosphate to increase fibroblast growth factor 23 production *in vitro*. *Journal of Endocrinology*.

[74] Ben-Dov I. Z., Galitzer H., Lavi-Moshayoff V. (2007). The parathyroid is a target organ for FGF23 in rats. *The Journal of Clinical Investigation*.

[75] Olauson H., Lindberg K., Amin R. (2013). Parathyroid-specific deletion of Klotho unravels a novel calcineurin-dependent FGF23 signaling pathway that regulates PTH secretion. *PLoS Genetics*.

[76] Rhee Y., Bivi N., Farrow E. (2011). Parathyroid hormone receptor signaling in osteocytes increases the expression of fibroblast growth factor-23 in vitro and in vivo. *Bone*.

[77] Gattineni J., Twombley K., Goetz R., Mohammadi M., Baum M. (2011). Regulation of serum 1,25(OH)_2_ vitamin D_3_ levels by fibroblast growth factor 23 is mediated by FGF receptors 3 and 4. *The American Journal of Physiology—Renal Physiology*.

[78] Arai-Nunota N., Mizobuchi M., Ogata H. (2014). Intravenous phosphate loading increases fibroblast growth factor 23 in uremic rats. *PLoS ONE*.

[79] Kawai M., Kinoshita S., Shimba S., Ozono K., Michigami T. (2014). Sympathetic activation induces skeletal *Fgf23* expression in a circadian rhythm-dependent manner. *The Journal of Biological Chemistry*.

[80] Smith R. C., O'Bryan L. M., Farrow E. G. (2012). Circulating *α*Klotho influences phosphate handling by controlling FGF23 production. *Journal of Clinical Investigation*.

[81] Kuro-o M., Matsumura Y., Aizawa H. (1997). Mutation of the mouse *klotho* gene leads to a syndrome resembling ageing. *Nature*.

[82] Takei Y., Yamamoto H., Sato T. (2012). Stanniocalcin 2 is associated with ectopic calcification in *α*-klotho mutant mice and inhibits hyperphosphatemia-induced calcification in aortic vascular smooth muscle cells. *Bone*.

[83] Yuan Q., Jiang Y., Zhao X. (2014). Increased osteopontin contributes to inhibition of bone mineralization in FGF23-deficient mice. *Journal of Bone and Mineral Research*.

[84] Sitara D., Kim S., Razzaque M. S. (2008). Genetic evidence of serum phosphate-independent functions of FGF-23 on bone. *PLoS Genetics*.

[85] Matsumura Y., Aizawa H., Shiraki-Iida T., Nagai R., Kuro-O M., Nabeshima Y.-I. (1998). Identification of the human klotho gene and its two transcripts encoding membrane and secreted klotho protein. *Biochemical and Biophysical Research Communications*.

[86] Shiraki-Iida T., Aizawa H., Matsumura Y. (1998). Structure of the mouse klotho gene and its two transcripts encoding membrane and secreted protein. *FEBS Letters*.

[87] Shalhoub V., Ward S. C., Sun B. (2011). Fibroblast growth factor 23 (FGF23) and *α*-klotho stimulate osteoblastic MC3T3.E1 cell proliferation and inhibit mineralization. *Calcified Tissue International*.

[88] Kawai M., Kinoshita S., Kimoto A. (2013). FGF23 suppresses chondrocyte proliferation in the presence of soluble*α*-klotho both in vitro and in vivo. *Journal of Biological Chemistry*.

[89] Faul C., Amaral A. P., Oskouei B. (2011). FGF23 induces left ventricular hypertrophy. *Journal of Clinical Investigation*.

[90] Angelin B., Larsson T. E., Rudling M. (2012). Circulating fibroblast growth factors as metabolic regulators—a critical appraisal. *Cell Metabolism*.

[91] Krejci P., Krakow D., Mekikian P. B., Wilcox W. R. (2007). Fibroblast growth factors 1, 2, 17, and 19 are the predominant FGF ligands expressed in human fetal growth plate cartilage. *Pediatric Research*.

[92] Wei W., Dutchak P. A., Wang X. (2012). Fibroblast growth factor 21 promotes bone loss by potentiating the effects of peroxisome proliferator-activated receptor *γ*. *Proceedings of the National Academy of Sciences of the United States of America*.

[93] Delrieu I. (2000). The high molecular weight isoforms of basic fibroblast growth factor (FGF-2): an insight into an intracrine mechanism. *FEBS Letters*.

[94] Claus P., Döring F., Gringel S. (2003). Differential intranuclear localization of fibroblast growth factor-2 isoforms and specific interaction with the survival of motoneuron protein. *Journal of Biological Chemistry*.

[95] Xiao L., Naganawa T., Lorenzo J., Carpenter T. O., Coffin J. D., Hurley M. M. (2010). Nuclear isoforms of fibroblast growth factor 2 are novel inducers of hypophosphatemia via modulation of FGF23 and KLOTHO. *The Journal of Biological Chemistry*.

[96] Wolf M. (2012). Update on fibroblast growth factor 23 in chronic kidney disease. *Kidney International*.

[97] Chen Z., Chen X., Xie J. (2013). Fibroblast growth factor 23 is a predictor of aortic artery calcification in maintenance hemodialysis patients. *Renal Failure*.

[98] Montford J. R., Chonchol M., Cheung A. K. (2013). Low body mass index and dyslipidemia in dialysis patients linked to elevated plasma fibroblast growth factor 23. *American Journal of Nephrology*.

